# Effect of Sirolimus/Metformin Co-Treatment on Hyperglycemia and Cellular Respiration in BALB/c Mice

**DOI:** 10.3390/ijms24021223

**Published:** 2023-01-08

**Authors:** Alia Albawardi, Dhanya Saraswathiamma, Charu Sharma, Abdulghani Elomami, Abdul-Kader Souid, Saeeda Almarzooqi

**Affiliations:** 1 Department of Pathology, College of Medicine and Health Sciences, United Arab Emirates University, Al Ain 15551, United Arab Emirates; 2 Department of Internal Medicine, College of Medicine and Health Sciences, United Arab Emirates University, Al Ain 15551, United Arab Emirates; 3 Division of Anatomy Pathology and Cytopathology, Tawam Hospital, Al Ain 15258, United Arab Emirates; 4 Department of Pediatrics, College of Medicine and Health Sciences, United Arab Emirates University, Al Ain 15551, United Arab Emirates

**Keywords:** sirolimus, metformin, cellular respiration, mTOR, hyperglycemia

## Abstract

Sirolimus (SRL) is widely used as an immunosuppressant to prevent graft rejection, despite the risk of impairing glucose metabolism. Metformin (MET) can reduce the detrimental effects of SRL in many patients, including diabetes and renal transplant recipients. Limited in vivo studies have reported on SRL and MET therapy, particularly in relation to cellular bioenergetics, glucose metabolism, and insulin resistance. Herein, we investigated the efficacy of SRL and MET co-treatment in BALB/c mice over 4 weeks. Balb/c mice (4–6 weeks old) were divided into four groups and injected intraperitoneally (i.p.) with water (control, CTRL), MET (200 µg/g), SRL (5 µg/g), or MET (200 µg/g) +SRL (5 µg/g) over a period of one month. We evaluated the body weight, food consumption rate, random blood glucose (BG), insulin levels, serum biochemistry parameters (ALT, Albumin, BUN, Creatinine), and histomorphology in all groups using standardized techniques and assays. All drug-treated groups showed a statistically significant decrease in weight gain compared to the CTRL group, despite normal food intake. Treatment with SRL caused elevated BG and insulin levels, which were restored with SRL + MET combination. Serum biochemical parameters were within the normal range in all the studied groups. SRL+ MET co-treatment decreased liver cellular respiration and increased cellular ATP levels in the liver. In the pancreas, co-treatment resulted in increased cellular respiration and decreased cellular ATP levels. Liver and pancreatic histology were unchanged in all groups. This study showed that co-treatment of SRL with MET alleviates hyperglycemia induced by SRL without any deleterious effects. These results provide initial insights into the potential use of SRL + MET therapy in various settings.

## 1. Introduction

Sirolimus (SRL) is an FDA-approved mammalian target of rapamycin (mTOR) inhibitor and immunosuppressant that is extensively used in transplant and oncology clinical settings [1,2]. SRL modulates the activity of the mTOR by binding to the FK binding protein [3]. This agent manipulates the immune system by inhibiting interleukin-2 signal transduction and by blocking T and B lymphocyte activation by cytokines, thus, ameliorating inflammation and cellular proliferation. SRL is predominantly metabolized in the liver, which serves as a substrate for Cytochrome P450 3A4 and P-glycoprotein [1,2] and induces metabolic dysfunction, including glucose intolerance and insulin resistance [4,5]. The primary side effect of chronic SRL therapy is the development of glucose intolerance through increased hepatic gluconeogenesis [6].

Metformin (1,1-dimethyl biguanide hydrochloride) is one of the most effective hypoglycemic agents against type 2 diabetes (T2D) [7]. MET is a potent regulator of cellular bioenergetics [8,9] and presumably refines several cellular energy conversion reactions, resulting in lower demand for hepatic gluconeogenesis and increased insulin sensitivity in the peripheral tissues. MET reduces hyperglycemia by inhibiting hepatic gluconeogenesis [10]. Furthermore, MET has been shown to elevate peripheral insulin sensitivity, increase peripheral glucose uptake, increase fatty acid oxidation, and reduce glucose absorption [5]. The use of MET does not result in hypoglycemia, as insulin secretion is unaffected [11]. MET functions by inhibiting the mTOR pathway [12]. In cultured rat astrocytes, MET diminishes complex 1-mediated mitochondrial respiration [13], while improved myocardial ATP levels were noted in mice with heart failure treated with MET [14].

Inhibition of mTOR is a promising strategy for preventing rejection after transplantation and autoimmune diseases. However, few studies have addressed the possibility of co-treatment with MET to reduce the deleterious effects of SRL in the treatment of diabetes and renal transplant recipients [15,16]. However, protocols have previously been adjusted to alleviate metabolic defects caused by SRL, including intermittent treatment or co-treatment with other drugs such as MET [5].

However, limited in vivo studies are available regarding co-treatment with SRL and MET therapy [4,17]. Furthermore, SRL and MET co-treatment has not been studied extensively in relation to cellular bioenergetics, glucose metabolism, and insulin resistance. As both SRL and MET are targets of the rapamycin complex 1 (mTORC1) [12], we aimed to determine the effect of in vivo co-treatment with SRL and MET on cellular bioenergetics and glucose metabolism. We further aimed to study the morphological changes in the liver and pancreas and serum biochemistry to determine its safety and effectiveness.

## 2. Results

### 2.1. Effect on Body Weight in Different Groups

**Figure 1:**
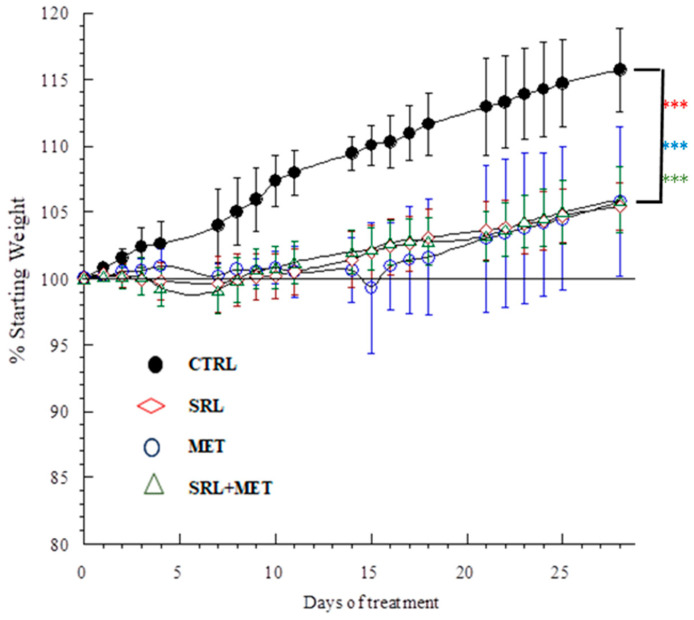
Weight changes in mice treated with H_2_O (control, CTRL), 5 µg/g SRL alone (SRL), 200 µg/g MET alone (MET), and the combination of 200 µg/g MET plus 5 µg/g SRL(SRL+MET). The results represent two separate experiments (3 mice per group in each experiment, a total of 24 mice). The values (mean ± SD) are percentages of daily weight divided by the starting weight. *** *p* < 0.001.

The effect of CTRL, SRL, MET, and SRL+ MET administration on the body weight of mice is shown in Figure 1. At the end of 4 weeks of treatment, all three treatment groups showed a significant reduction in weight gain compared to the CTRL group. Body weight changes (mean ± SD) in the groups of mice receiving different drug treatments were expressed as percentages, calculated as the mouse weight on each day divided by the starting mouse weight). The absolute starting body weight (mean ± SD, *n* = 6) of CTRL, SRL, MET, and SRL + MET groups were 23.2 ± 3.9 g, 24.6 ±1.8 g, 24.45 ± 2.5 g and 23.7 ± 2.4 g respectively. Compared to the CTRL group, all three treated groups showed a significant reduction in percentage weight gain (*** *p* < 0.001). There was no significant difference in the percentage of weight gain among the three treated groups.

### 2.2. Effect of Food Consumption Rate in Different Groups

**Figure 2:**
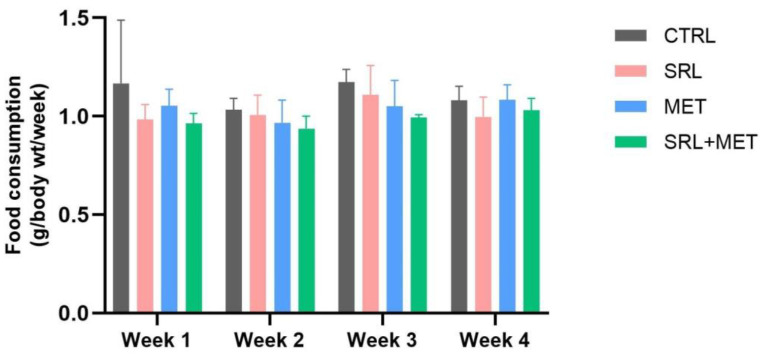
Weekly food intake (g/g body weight/week), in Control, MET alone, SRL alone, and MET-SRL mice groups during the treatment period. The results are representative of two independent experiments. Data represents means ± SEM (standard error of the mean).

To determine whether the diminished weight gain in the treated groups was due to reduced appetite, food consumption was determined (Figure 2). No significant reduction in food consumption rate was observed in any of the drug treatment groups compared to the controls.

### 2.3. Effect on Hyperglycemia and Insulin Secretion in Different Groups

**Figure 3:**
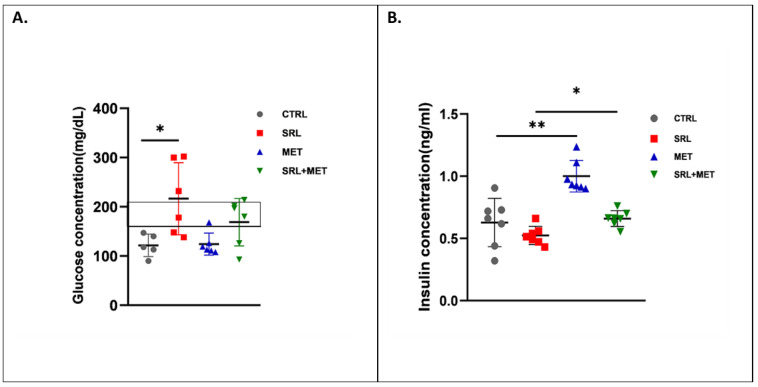
Serum glucose and insulin levels of Balb/C. (**A**) Serum glucose levels in mice after 4 weeks of treatment with Water (CTRL)or SRL MET orSRL+MET therapy. Random blood glucose levels in the prediabetic range are represented by the box. (**B**) Serum Insulin levels in mice after 4 weeks of treatment with CTRL, MET or SRL alone, or MET +SRL (*n* = 4–6 per group), Data are means ± SD. * *p* < 0.05, ** *p* < 0.01.

The SRL-treated group showed increased random BG levels in mice compared to the CTRL group after 4 weeks of treatment (216.3 ± 66.9 mg/dL, * *p* < 0.05). Although the difference in BG levels was insignificant (*p* = 0.212) between SRL alone and SRL+MET group, the levels were in the pre-diabetic range (168.7 ± 44.0 mg/dL) in the SRL+MET group (Figure 3A).

We also measured the differences in insulin secretion, and it was noted that MET treatment caused an increase in the secretion of insulin (Figure 3B). Mice that received MET alone showed an increase in insulin concentration of 1.13 ± 0.22 ng/mL compared to the CTRL group (*p* < 0.01). The SRL group of mice produced lower levels of insulin than the CTRL group, but the difference was not statistically significant. The insulin concentration was 0.62 ± 0.22 ng/mL in the CTRL group, which was reduced to 0.53 ng/mL ± 0.17 in the SRL-treated group. Co-treatment with MET improved insulin concentration in the SRL+MET group of mice (*p* < 0.05). The decrease in insulin concentration in the SRL alone group was rescued in SRL, MET co-treatment group (0.67 ± 0.25 ng/mL, * *p* < 0.05).

### 2.4. Detection of Pattern and Number of β and α Cells in Pancreas

**Figure 4:**
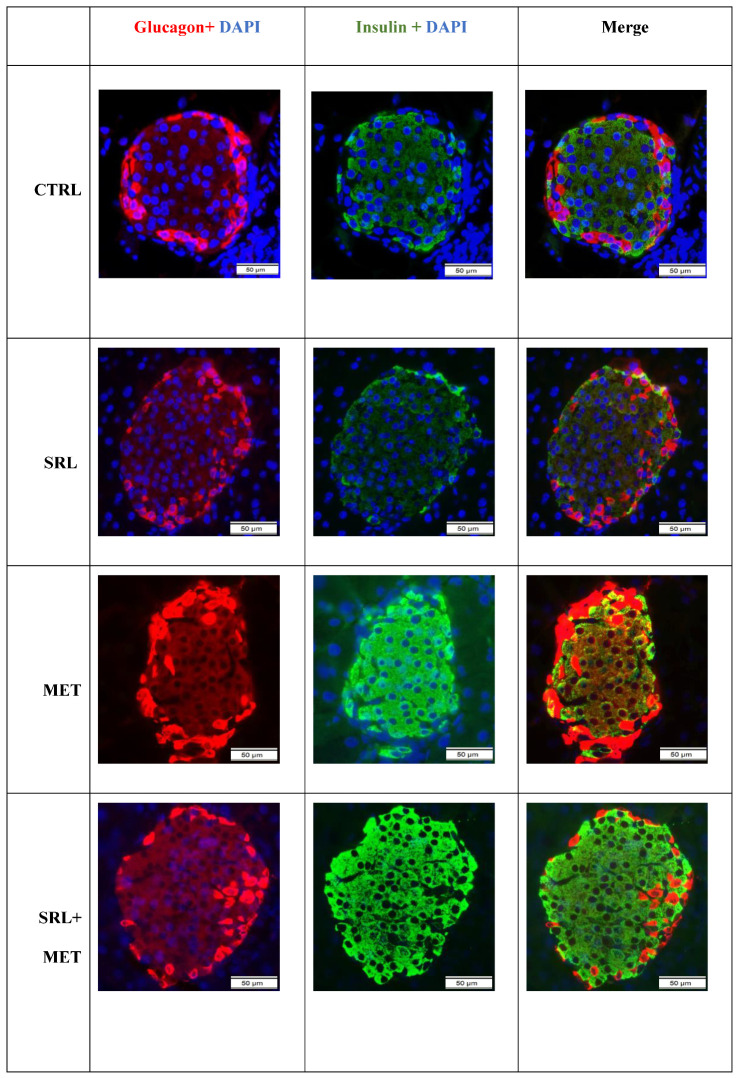
Immunofluorescence double labelling for insulin (red) and glucagon (green) in pancreatic islet cells from mice after one month of treatment with Water (CTRL), SRL alone, MET alone, and SRL+MET co-treatment groups. Pancreatic sections from Balb/C mice double immunostained for insulin (green) and glucagon (red), with nuclei visualized by DAPI staining. Magnification of 40X. *n* = 6 mice/group.

**Figure 5:**
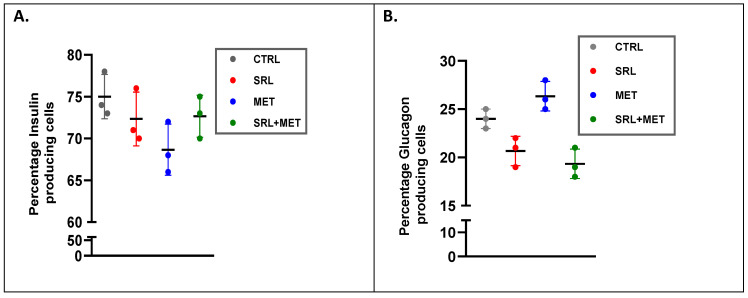
The percentage distribution of (**A**) insulin and (**B**) glucagon-positive cells in mice pancreatic islets of different treatment groups (CTRL, SRL, MET, and SRL+MET). There was no significant difference was noted in the pattern of distribution of insulin and glucagon-positive cells in any of the studied groups(*n* = 3).

The percentage and distribution pattern of insulin-producing β cells and glucagon-producing α cells in the pancreatic tissue was studied using the immunofluorescence (IF) technique (Figure 4). We did not observe any significant differences in distribution or percentage of insulin and glucagon-producing cells in CTRL, SRL, MET, or SRL+MET groups. The percentage of insulin-producing β-cells is 50–70%, and glucagon-producing α-cells is 20–30% in normal pancreatic cells [18]. This percentage has remained the same in all treatment groups (Figure 5).

### 2.5. Effect on Cellular Respiration in Different Groups

Cellular respiration rate and ATP content were measured in the liver and pancreatic tissues to study any differences in glucose metabolism. Graphical representations of cellular respiration in different organs are shown in our previous publications [19]. In the present study, we observed a trend of decrease in cellular respiration among all the treatment groups; however, this decrease was more pronounced in the group treated with a combination of both SRL and MET (*p* = 0.180, Table 1). Pancreatic cellular respiration showed a significant increase with SRL alone and in combination with SRL + MET (*p* = 0.045, Table 1). Liver and pancreas cellular ATP contents in the MET-treated mice group were similar to that of the CTRL. However, liver cellular ATP contents in the SRL-treated group and SRL+MET group were significantly higher than CTRL (*p* = 0.016, Table 1). In contrast, ATP contents in the pancreas did not differ significantly between drug-treated groups and CTRL groups (Table 1).

### 2.6. Biochemical Changes in Different Groups

**Figure 6:**
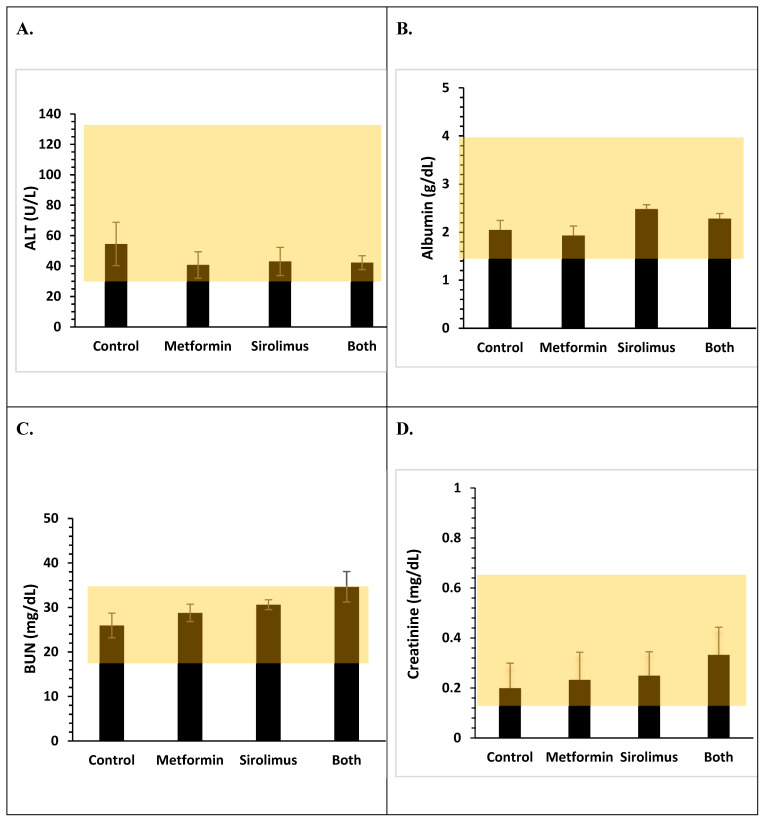
Clinical chemistry parameters of Balb/C mice after one month of treatment with water or MET alone or SRL alone or MET + SRL therapy following which blood was collected and analysed for the indicated parameters. (**A**) Serum alanine aminotransferase, ALT(U/L)) levels. (**B**) Serum Albumin (g/dL) levels. (**C**) Serum blood urea nitrogen, BUN (mg/dL). (**D**) Serum creatinine (mg/dL) levels. The shaded box in each graph represents the normal range for that particular parameter. The results are representative of two independent experiments.

To assess the potential toxicity/complications associated with SRL + MET co-treatment, we sacrificed mice at the end of 4 weeks of treatment, and their blood was collected for biochemical analysis. Serum creatinine, BUN, ALT, and albumin levels were measured. We did not observe any significant differences in the levels of creatinine, BUN, ALT, or albumin in all treatment groups as compared to control (*p* > 0.05, Figure 6).

### 2.7. Histological Examination among Different Groups

**Figure 7:**
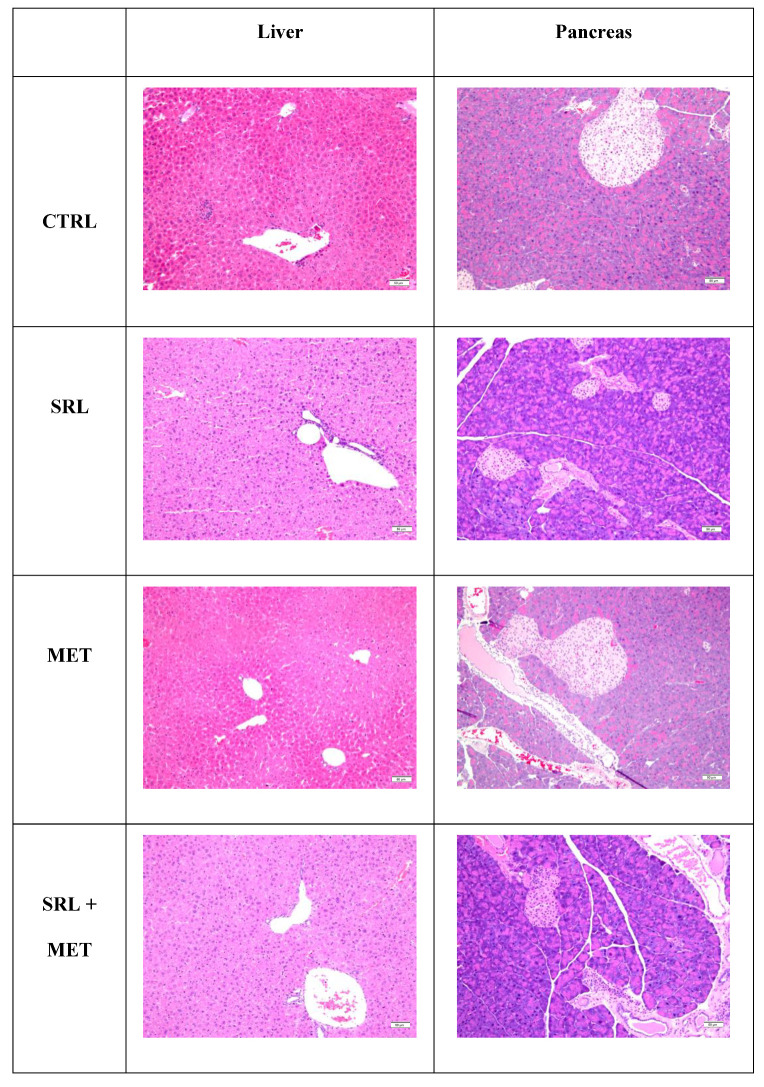
Representative histology of the liver and pancreas in mice untreated and mice treated with water (CTRL), 5 µg/g of SRL alone, 200 µg/g of MET alone and SRL+MET cotreatment. Liver architecture is preserved and hepatocytes are intact with the absence of vacuolization, apoptosis, and parenchymal inflammation (hematoxylin and eosin, 20x). The pancreas also reveals intact exocrine glands and islet cells in all studied groups (hematoxylin and eosin, 20x).

**Figure 8:**
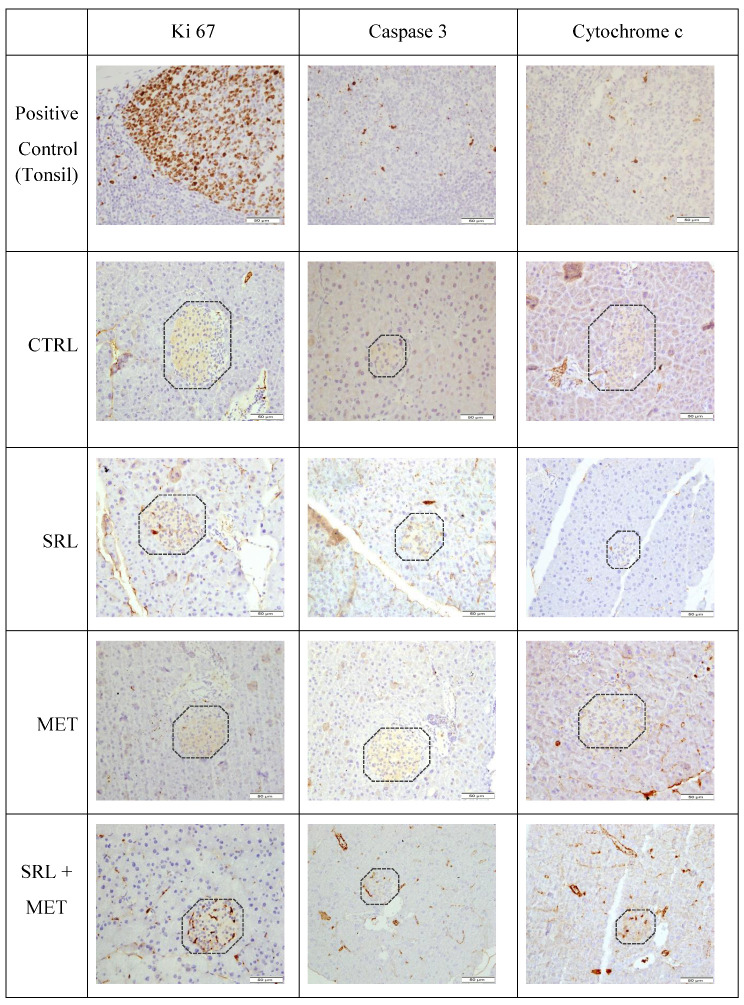
Immunohistochemical staining for Ki 67, caspase-3, and cytochrome C in pancreas sections of mice treated with water (CTRL), 5 µg/g of SRL alone, 200 µg/g of MET alone and SRL+MET cotreatment for 4 weeks. Magnification 40X.

Histological examination of the liver revealed preserved architecture in all of the studied groups. Pancreatic morphology was also unaltered in the three treatment groups compared to CTRL (H&E staining, Figure 7). No morphological alterations in hepatocytes, such as apoptosis, vacuolization, and inflammation, were detected in any of the mice. Immunohistochemical analysis of Ki-67, Caspase 3, and Cytochrome c was performed to confirm the observations from H&E in both liver and pancreatic tissues. Ki-67 staining was absent in islets cells, or only a single positive cell was noted in the different conditions. There was no significant difference in staining in the different groups. The studied groups were also stained for apoptotic markers caspase 3 and Cytochrome c, which again did not disclose any difference in the different groups. (Figure 8). Immunohistochemical analysis on liver tissue for Ki 67, caspase 3, and Cytochrome c also did not show any difference in any of the studied groups (Supplementary Figure S1).

## 3. Materials and Methods

### 3.1. Reagents and Solutions

MET (molecular weight: 129.164 g/mol) was obtained from Toronto Research Chemicals (North York, Canada); a 100 mM solution was prepared in H_2_O and stored at −20 °C. Rapamycin (SRL) was procured from MedChem Express (Princeton, NJ, USA). For preparation, SRL was dissolved in dimethyl sulfoxide (DMSO) to a stock concentration of 50 µg/µL (55 mM) and kept at −20 °C in small aliquots. Immediately before use, aliquots were diluted to 0.5 µg/µL in dH_2_O. The Pd (II) complex (meso-tetra-(4-sulfonatophenyl)-tetrabenzoporphyrin (Pd phosphor) was purchased from Porphyrin Products (Logan, UT, USA), and a stock solution (2.5 mg/mL = 2 mM) was prepared in distilled water and frozen at −20 °C until use. A complete protease inhibitor cocktail was purchased from Roche (IN, USA); one tablet was dissolved in 1.0 mL H_2_O and stored at −20 °C. RPMI 1640 medium and other reagents were purchased from Sigma-Aldrich (St. Louis, MO, USA). The cellular respiration reaction mixture contained 3 reagents-1.0 mL of RPMI medium, 3 µM Pd phosphor, and 0.5% fat-free albumin.

### 3.2. Mice and Treatment

Adult BALB/c mice, 6–8 weeks old, were housed at 22 °C, 60% humidity, and 12-h light-dark cycles. Mice were provided filtered water and rodent chow ad libitum. Mice food consumption was determined by subtracting leftover weights from initial known weights. This study was approved by the Animal Ethics Committee of the College of Medicine and Health Sciences, UAE University (#A29-13). Mice received i. p. injections of distilled water (dH2O, CTRL group), MET alone (200 µg/g), SRL (5 µg/g), or co-treatment (MET 200 µg/g +SRL 5 µg/g) for five uninterrupted days every week for four weeks. SRL and MET doses were selected based on previous mouse studies and correspond to the mouse equivalent of the maximum tolerated dose (MTD) [20,21]. The MET solution was freshly prepared before use by dilution in distilled water. The SRL solution was prepared immediately before use in dH_2_O. Percent weight changes (mean ± SD) were defined as the daily weight divided by the starting weight and used as a surrogate marker for drug activity. Fragments of the liver and pancreas were collected at the end of in vivo treatment and processed to determine cellular respiration, cellular ATP, and histology.

### 3.3. Mice Tissue Collection and Processing

Anesthesia was induced with 10 µL/g urethane (25% w/v) administered intraperitoneally for anesthesia. Fragments of the liver and pancreas were then quickly excised with a sterile scalpel and immersed in 10 mL ice-cold RPMI medium supplemented with 10 µL of complete protease inhibitor cocktail solution and saturated with 95% O2 and 5% CO2. One tissue piece was immediately immersed in ice-cold 2% trichloroacetic acid and processed for ATP determination. Another piece of tissue was immediately placed in an oxygen-measuring vial to determine the rate of cellular respiration at 37 °C.

For histological analysis, tissue fragments were fixed in 10% formalin and embedded in paraffin. Section 3, Section 4 and Section 5 µm-thickness were then cut and stained with hematoxylin and eosin (H&E). Section 4 and Section 5 µm thickness were placed on silane-coated microscope slides to perform Immunofluorescence and Immunohistochemistry analysis.

For insulin glucagon co-staining using the Immunofluorescence technique, primary antibodies against insulin (1:2000, ICBTACL5, Invitrogen, Waltham, MA, USA) and glucagon (1:2500, ab92517, Abcam, Cambridge, United Kingdom) were applied for incubation overnight at 4 °C. Alexa fluor-conjugated secondary antibodies were applied for 1 h at room temperature (1:500 dilution, A11037, and A11029, Invitrogen, Karlsruhe, Germany). Nuclei were stained with DAPI, and sections were covered using Immunomount^®^ (Shandon, Pittsburgh, PA, USA) and observed using a Nikon Eclipse Ni fluorescent microscope (Nikon Instruments Europe BV, Cells 2021, 10, 1981 4 of 20 Tripolis 100, Amsterdam, Netherlands). The images were analyzed using ImageJ software.

Immunohistochemistry staining was performed for Ki-67 (1:200 dilution; DAKO, Santa Clara, United States), Caspase-3 ((1:200 dilution; Cell Signaling Technology, Danvers, MA, USA), and Cytochrome c (1:200 dilution; Cell Signaling Technology, Danvers, MA, USA). The rabbit- and mouse-specific secondary antibodies (1:50 dilution; Cell Signaling, USA) followed by horseradish peroxidase/3,3′-diaminobenzidine detection immune-histochemistry kit (Vector Laboratories, Burlingame, CA, USA).

### 3.4. Biochemical Analysis/Serum Biochemical Analysis

Biochemical analysis of the blood, including glucose, creatinine, BUN, ALT, and albumin levels, was performed using standard laboratory methods. A commercially available kit was purchased from Roche (Basel, Switzerland) using a blood chemistry analyzer (Roche COBAS, Switzerland).

### 3.5. Serum Insulin Measurement

Serum insulin levels were determined using an ultrasensitive rat insulin ELISA kit (Mercodia, Uppsala, Sweden), according to the manufacturer’s instructions. A standard curve was constructed using standards of known concentrations provided with the kit. The insulin concentration in each sample was then interpolated from the standard curve. Insulin concentration was measured at a wavelength of 450 nm using an EMax Plus microplate reader (Molecular Devices, San Jose, CA, USA).

### 3.6. Cellular Respiration

A phosphorescence oxygen (O_2_) analyzer was used to determine the rate of cellular mitochondrial O_2_ consumption [22,23,24] in the liver and pancreatic tissues. Briefly, tissue fragments were placed in oxygen-measuring vials containing 1.0 mL RPMI medium supplemented with 0.5% fat-free bovine albumin and 3 µM Pd phosphor. The vials were sealed from air and dissolved O2 was measured at 37 °C as a function of time. The rate of cellular respiration (kc, µM O2 min-1) was the negative of the slope of [O2] vs. time (t). the value of kc was obtained by normalizing cellular respiration value (k) by the tissue weight (mg). The oxygen was detected using Pd phosphor with an absorption maximum of 625 nm and an emission maximum of 800 nm. The samples were exposed to 600 flashes/min, peaking at 625 nm, filtered at 800 nm, and detected using a Hamamatsu photomultiplier tube. The phosphorescence decay rate (1/τ) was exponential and linear with the dissolved oxygen concentration:1/τ = 1/τ o + kq [O2], (1/τ = the phosphorescence decay rate in the presence of O2, 1/τ o is the phosphorescence decay rate in the absence of O_2_, and kq is the second-order oxygen quenching rate constant in s-1 • τ M-1).

### 3.7. Cellular ATP

Tissue fragments were homogenized in ice-cold 2% trichloroacetic acid (freshly prepared), and the supernatant was stored for later use at −20 °C. Before ATP determination, the supernatant was neutralized with tris-acetate (100 mM) and 2 mM ethylenediaminetetraacetic acid (pH 7.75). The results are expressed as μmol ATP per mg dry pellet weight. Measurements were performed using the Enliten ATP Assay System (Bioluminescence Detection Kit and Glomax Luminometer (Promega, Madison, WI, USA), as previously described [23].

## 4. Statistical Analysis

The results are expressed as either the mean ± SD or mean ± SE, as indicated. Statistical analysis was performed using one-way ANOVA or unpaired t-test. GraphPad Prism version 9 software (San Diego, CA, USA) was used for the statistical analysis. Differences were considered significant at * *p* < 0.05, ** *p* < 0.01, *** *p* < 0.001, or **** *p* < 0.0001. Cellular bioenergetics data were analyzed using SPSS statistical package (version 29).

## 5. Discussion

In this study, we determine the effect of MET co-treatment in alleviating the SRL-induced side effects for a duration of four weeks in Balb/C mice.

It is well known that SRL affects animal weight, and some studies have also reported the underlying mechanisms [25,26,27]. One study suggested that SRL treatment is associated with increased mTOR signaling in hypothalamic neurons, leading to changes in the central homeostatic sensor of body-weight control [28]. In the present study, there was a reduction in percentage weight gain after SRL or MET treatment compared to CTRL, and this effect was not intensified with SRL + MET co-treatment. The reduction in percentage weight gain after SRL/MET treatment was similar to that observed with SRL + MET. This lack of an additive effect may be due to the fact that both SRL and MET target the rapamycin complex 1 (mTORC1) receptors [12]. We also observed that all three treated groups consumed less food than CTRL, although it was not significant. This anorectic effect, along with increased energy expenditure, might be the reason behind the reduced percentage of weight gain in the animals. Studies have previously reported inconsistent results regarding the effect of SRL/MET on food intake [25,26,27].

SRL-induced hyperglycemia was evident in the SRL alone group compared to CTRL. The mice showed significantly increased BG levels in comparison with the CTRL group, which may be due to the disruption of glucose homeostasis by SRL therapy. SRL+MET co-treatment significantly lowers BG levels in mice after 4 weeks suggesting MET can counteract the adverse effects of SRL. It has been well established clinically that MET, unlike SRL, lowers gluconeogenesis [6,29]. The antagonistic effect of these two drugs on gluconeogenesis may explain the improvements in glucose homeostasis in the group that received SRL+ MET co-treatment. Additionally, present findings are in accordance with recent studies suggesting that metabolic dysfunction caused by SRL can be dissociated from its mTOR inhibitory effects, either through pharmaceutical therapy or through alternative treatments [5,30,31,32].

Serum insulin levels were examined in all treatment groups as it plays a major role in maintaining glucose homeostasis [33]. Reduced serum insulin levels were observed in the SRL group, but there was a significant increase in serum insulin levels after SRL + MET treatment. Various studies have reported that MET improves insulin secretion and sensitivity by enhancing the insulin-mediated receptor tyrosine kinase activity, which activates the post-receptor insulin signaling pathways [34,35,36,37]. MET improves both hepatic and peripheral insulin sensitivity, exerting both direct and indirect effects on the liver and muscles [38,39,40]. In the present study, we also observed that MET increased insulin secretion, thereby reducing BG levels to the normal range in the SRL + MET co-treatment group. A previous study has reported that SRL-induced cell dysfunction does not affect cell proliferation or viability [41]. Similarly, our study also showed that despite differences in circulating insulin concentrations, there was no significant difference in the percentage of insulin- and glucagon-producing cells in any treated groups.

As mTOR influences the flux of nutrients into mitochondria, we also studied the effect of SRL+ MET co-treatment on cellular bioenergetics, including cellular respiration and quantification of ATP levels, in the liver and pancreas. Cellular bioenergetics encompasses biochemical processes involved in energy conversion. More specifically, cellular respiration involves the delivery of O_2_ to the mitochondria, breaking down reduced metabolic fuels, and passing electrons to O_2_ to form H_2_O (oxidation) [20]. The effect of cellular bioenergetics in the liver has been established in various animal models treated with SRL [23,42]. In the present study, we observe a decreased cellular respiration in liver tissues for the group receiving SRL+MET co-treatment. Conversely, cellular ATP levels in liver tissue were increased in the SRL+MET co-treatment group (consistent with the hypothesis of metabolic/mitochondrial fitness). This difference might be due to the natural response of mitochondrial activities in a high-glucose environment. Studies have shown that both hyperglycemia and hyperlipidemia treatment significantly increased respiration, decreased ATP content, lowered mitochondrial membrane potential, and increased mitochondrial volume, signs that indicate mitochondrial uncoupling and dysfunction [43]. AlsoMoreover, it has already been established that mTOR inhibitors impair oxygen respiration rates both in vitro and in vivo [42]. However, in pancreas SRL alone and SRL + MET co-treatment resulted in increased cellular respiration and decreased cellular ATP. This may suggest that modulation of cellular respiration by SRL is organ-specific [41].

To study the safety of SRL and MET alone and co-treatment, we also studied the biochemical and morphological changes within the groups. We did not observe any significant changes in the levels of BUN, ALT, and albumin in any of the treatment groups, and all the parameters were within the normal range, suggesting the safety of this co-treatment.

Histological analysis revealed no deleterious morphological effects on both liver and pancreas tissues after 4 weeks of treatment in any of the studied groups. Both proliferative and apoptotic markers showed minimal positivity in all the treated and CTRL groups. This may suggest that all the metabolic changes were happening at the molecular level.

## 6. Limitations

This study was conducted over a period of 4 weeks, and no structural or morphological changes were observed among the studied groups. Further studies are planned to examine the structural and ultrastructural changes induced by chronic treatment to better understand the mechanisms. Advanced research is needed to determine whether the mitochondrial ultrastructure is affected by any of these drugs.

## 7. Conclusions

This study evaluates the potency, effectiveness, and safety of MET co-treatment in alleviating SRL-induced side effects. SRL-induced hyperglycemia is improved by the addition of MET to the treatment regimen. The declined insulin secretion caused by the SRL is also improved by the introduction of MET therapy. Overall, the results of our study demonstrate that MET is certainly beneficial when used in combination with SRL. It contributes to the growing evidence suggesting alternative treatment regimens with SRL may be useful for maximizing its effects with minimal side effects.

## Figures and Tables

**Table 1:** Liver and pancreas cellular respiration and ATP content in mice treated with water (CTRL), sirolimus alone (SRL), metformin alone (MET), or a combination of SRL and MET(SRL + MET).

	CTRL	SRL (5 μg/g)	MET (200 μg/g)	SRL (5 μg/g) + MET (200 μg/g)	*p*
*Cellular Respiration (µM O_2_min^−1^mg^−1^)*					
Liver	0.36 ± 0.07	0.32 ± 0.02	0.31 ± 0.07	0.26 ± 0.05	0.180
Pancreas	0.29 ± 0.07	0.38 ± 0.05 *	0.30 ± 0.00	0.37 ± 0.05 *	0.045
*Cellular ATP (pmol mg^−1^)*					
Liver	1656 ± 220	2000 ± 208 *	1693 ± 657	2371 ± 228 *	0.016
Pancreas	2086 ± 709	1920 ± 572	1920 ± 560	1689 ± 421	0.724

Values are mean ± SD (*n* = 3–8 mice per group). Values of *p* are asymptotic 2-tailed significance (nonparametric Kruskal-Wallis test, four independent samples). * *p* < 0.05.

## Data Availability

Data used in this manuscript is available within the article or supplementary material.

## Supplementary Materials

The following supporting information can be downloaded at: https://www.mdpi.com/article/10.3390/ijms24021223/s1.
