# Supplementary material for: Effect of Sirolimus/Metformin Co-Treatment on Hyperglycemia and Cellular Respiration in BALB/c Mice
Source: Int J Mol Sci. 2023 Jan 8;24(2):1223. doi: 10.3390/ijms24021223 (PMC9866855; doi:10.3390/ijms24021223)
Supplement: Supplementary file 1 [file ijms-24-01223-s001.zip › ijms-1999375-supplementary.pdf]

Supplementary figure

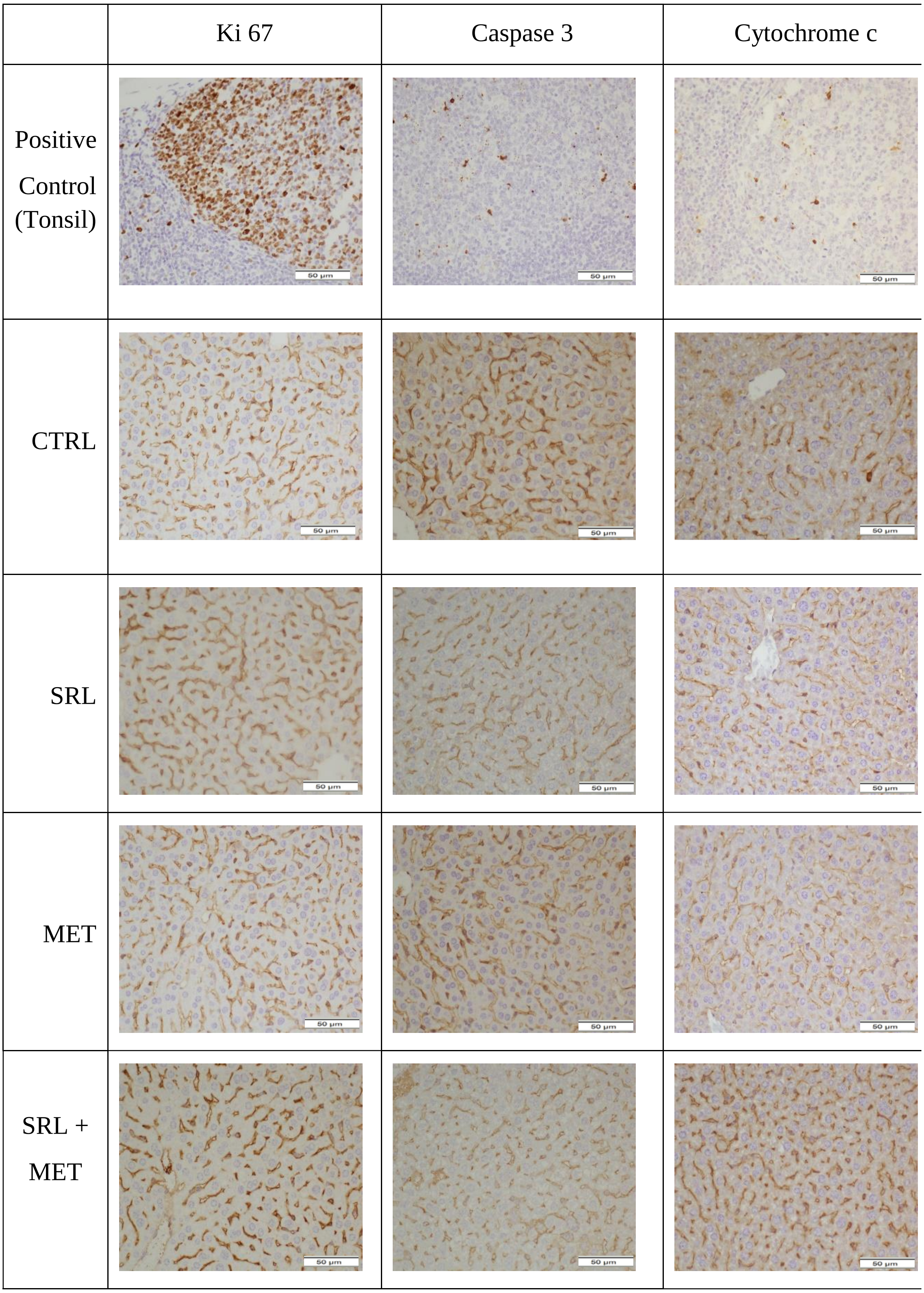

Figure S1. Immunohistochemical staining for Ki 67, caspase-3, and cytochrome C in liver sections of mice that undergone 4 weeks of treatment with water (CTRL), 5 µg/g of SRL alone, 200 µg/g of MET alone and SRL+MET cotreatment. Magnification 40X.
